# Sononeoperfusion: a new therapeutic effect to enhance tumour blood perfusion using diagnostic ultrasound and microbubbles

**DOI:** 10.1186/s40644-023-00545-y

**Published:** 2023-03-23

**Authors:** Najiao Tang, Jiawei Tang, Junhui Tang, Qiong Zhu, Xiaoxiao Dong, Yi Zhang, Ningshan Li, Zheng Liu

**Affiliations:** Department of Ultrasound, Xinqiao Hospital, Army Medical University, 83 Xinqiao Street, Chongqing, 400037 PR China

**Keywords:** Sononeoperfusion, Tumour, Diagnostic ultrasound, Microbubble, Doxorubicin

## Abstract

**Background:**

Hypoperfusion or resultant hypoxia in solid tumours is a main reason for therapeutic resistance. Augmenting the blood perfusion of hypovascular tumours might improve both hypoxia and drug delivery. Cavitation is known to result in microstreaming and sonoporation and to enhance drug diffusion into tumours. Here, we report the ability to enhance both tumour blood perfusion and doxorubicin (Dox) delivery using a new sononeoperfusion effect causing a cavitation effect on tumour perfusion in subcutaneous Walker-256 tumours of rats using ultrasound stimulated microbubble (USMB).

**Methods:**

To induce the sononeoperfusion effect, USMB treatment was performed with a modified diagnostic ultrasound (DUS) system and SonoVue® microbubbles. The therapeutic pulse was operated with a peak negative pressure of 0.26 to 0.32 MPa and a pulse repetition frequency (PRF) of 50 Hz to 2 kHz. Contrast-enhanced ultrasound (CEUS) was used for tumour perfusion assessment.

**Results:**

The USMB treatment of 0.26 MPa and 1 kHz could significantly enhance tumour perfusion with a 20.29% increase in the CEUS peak intensity and a 21.42% increment in the perfusion area for more than 4 hours (*P* < 0.05). The treatment also increased Dox delivery to tumours by approximately 3.12-fold more than that of the control (*P* < 0.05). Furthermore, ELISAs showed that vasodilators and inflammatory factors increased 4 hours after treatment (*P <* 0.05), suggesting that the inflammatory response plays an important role in the sononeoperfusion effect.

**Conclusion:**

The USMB-induced sononeoperfusion effect could significantly enhance the blood perfusion of Walker-256 tumours and promote drug delivery. It might be a novel physical method for overcoming the therapeutic resistance of hypoperfused or hypoxic tumours.

**Supplementary Information:**

The online version contains supplementary material available at 10.1186/s40644-023-00545-y.

## Background

Due to the upregulation of angiogenesis in solid cancers, tumour cells often grow more rapidly than the cells that form blood capillaries [1, 2]. Proliferation and immaturity force vessels away from tumour cells, leading to vascular density reduction and a poorly organized vessel architecture [3, 4], irregular tumour blood flow [5–8] and the compression of vessels by cancer cells [9, 10]. Therefore, hypoperfusion or hypovascularization tends to develop in solid tumours and has been considered a main factor for tumour hypoxia. Hypoperfusion and resultant hypoxia are strongly related to therapeutic resistance, such as in pancreatic cancer and ovarian cancer [11–13].

Another important factor for the response to chemotherapy in tumours is the distance from tumour vessels to tumour cells. At approximately 40–50 μm away from the blood vessel, the doxorubicin (Dox) concentration drops to half of its perivascular concentration. Furthermore, the average distance from tumour blood vessels to hypoxic tissue is 90 to 140 μm [14]. Therefore, tumour cells that are distant from blood vessels might be exposed to low concentrations of drug and a hypoxic state [10].

However, it is difficult to enhance tumour perfusion because vasoactive drugs may create vascular steal, leading to a decrease in tumour perfusion [15]. Previously, Xie and Lindner found that low intensity ultrasound (US) with a high mechanical index (MI) of 0.6–1.3 and microbubbles could stimulate and enhance myocardial or muscular perfusion, which was later called “sonoreperfusion” [16–18]. Sonoreperfusion may be connected with the increase in ATP and the purinergic pathway [19]. In our previous studies, we occasionally found that low MI diagnostic ultrasound (DUS) combined with microbubbles could enhance tumour blood perfusion and increase the perfusion area of solid tumours, such as PANC-1 pancreatic cancer and MC38 colon cancer in mice [20, 21]. This is a very interesting phenomenon, not only because the DUS intensity for this treatment was extremely low and within FDA and IEC guidelines, but also because it only requires low MI emission under 0.5, which is much lower than that of the MI needed for muscular sonoreperfusion [18]. This US-stimulated perfusion enhancement effect of solid tumours might be a novel and noninvasive method to overcome tumour hypoxia, a major obstacle to therapy.

Considering the low intensity, low MI and tumour perfusion response features of the treatment, we named the effect “sononeoperfusion”, representing US, neoplasm and perfusion improvement. Sonoreperfusion usually refers to US-stimulated myocardial or muscular blood reperfusion effects. However, tumour vascular construction is chaotic, disorganized, immature, dysfunctional and mechanically vulnerable, which is quite different from normal developed vessels [22, 23].

Therefore, the aim of this study was to investigate the proper parameters, Dox delivery and related mechanism for sononeperfusion. The parameters included the MI or acoustic pressure and the pulse repetition frequency (PRF). Cytokines such as vasodilators and inflammatory factors were tested to explain the mechanism.

## Methods

### DUS system and acoustic detection

**Fig. 1:**
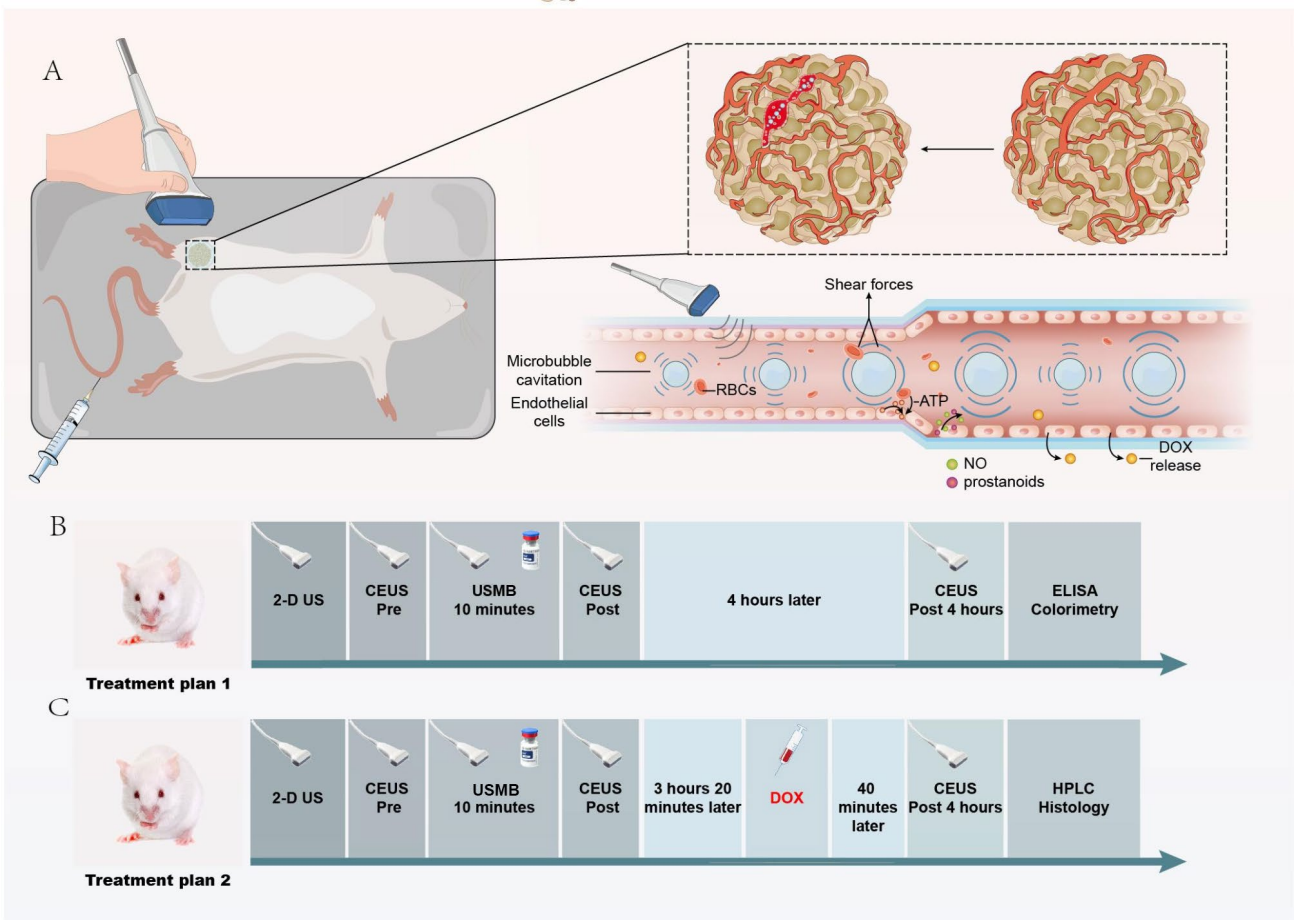
Schematic illustration of the USMB treatment in rats. A: Tumour perfusion enhancement is related to vasodilator release and promotes Dox uptake. B: Treatment plan 1: 56 SD rats received USMB treatment for 10 minutes and CEUS imaging at baseline, immediately after and 4 hours later. The control group received sham DUS. C: Treatment plan 2: Another 25 rats were enrolled for the Dox delivery study. The animals were treated with the selected USMB parameters and sham. After treatment, Dox solution was injected at approximately 3 hours 20 minutes.

A commercial DUS system (VINNO70, VINNO Technology Co. Ltd., Suzhou, China) connected to an X4-12 L linear array transducer was used for both therapeutic US exposure and US imaging. The system was equipped with contrast bubble imaging (CBI), integrated contrast-enhanced ultrasound (CEUS) imaging software and flash mode for microbubble destruction. The flash mode was specifically modified to deliver customized pulse sequences for regulating microbubble cavitation or so-called ultrasound stimulated microbubble (USMB), named Vflash. The Vflash pulses can be operated with adjustable frequency, MI, pulse length (PL), PRF and destruction (on)/replenish (off) time as described previously [24]. In addition, the Vflash US beams can be weakly focused to a trapezoid region of interest (ROI) using the electronic focusing method (Fig. 1). The size of the ROI is also adjustable and can cover the tumour body, similar to a colour doppler sample volume.

The peak negative pressure (PNP) within a designated ROI of 1 × 1 cm was measured by a membrane hydrophone (HMB-0500, ONDA Corp., Sunnyvale, CA, USA) positioned 2 cm away from the probe surface. The probe was placed above the hydrophone separated with degassed water in a sink (AIMS III, ONDA Corp., Sunnyvale, CA, USA).

### Microbubbles

SonoVue® microbubbles (Bracco Sine Pharmaceutical Corp., Ltd., Shanghai, China) are a commercially available US contrast agent that contains 10^8^ mL^− 1^ microbubbles with a mean diameter range from 2.0 to 4.0 μm after preparation in 4.0 mL of saline solution. The SonoVue® suspension was then used in both CEUS as an US contrast agent and in therapeutic US as cavitation nuclei. CEUS is a reliable method to assess tumour perfusion. According to the EFSUMB guidelines, the mean time-intensity curves within the tumours after bolus injection of a contrast agent were qualified to reflect microbubble wash-in and washout, thus representing the condition of tumour perfusion [25].

### Animal model and experimental design

A total of 81 Sprague‒Dawley (SD) rats bearing subcutaneous Walker-256 tumours were used. The tumour model was made by injecting 0.2 mL of Walker-256 cell suspension (approximately 1 × 10^7^/mL) into the inner thigh of the rat. Then, the model was included in the study when the tumour size reached approximately 1 cm in diameter. All of the animal experimental procedures were approved by the Institutional Animal Care and Use Committee of the university.

Among the 81 SD rats, 56 rats were randomly divided into seven groups, including six experimental groups (A-F) according to different treatment parameter combinations and one control group. The treatment parameters of all groups are illustrated in Table 1. All of the treatments were performed with an X4-12 L linear array transducer operating at a central frequency of 4 MHz. Two PNP outputs of 0.26 MPa and 0.32 MPa (equal to MI values of 0.13 and 0.16, respectively), which were measured by the hydrophone, were selected to test the PNP variable in the experimental groups. Under the fixed PL of 10.5 cycles, we selected 50 Hz, 1 and 2 kHz as three PRF variables (Table 1).

In treatment plan 2 (Fig. 1C), Dox served as a chemotherapeutic agent because it was detectable by fluorescent imaging and quantified by high-performance liquid chromatography (HPLC). For Dox delivery, another 25 rats were randomly divided into one experimental group (n = 14) and one control group (n = 11).

**Table 1:** The treatment parameters of all groups.

Groups	US parameters				Dox injection(mg/kg)	
	Frequency (MHz)	PNP (MPa)	PL (cycles)	PRF (Hz)		
Control	-	-	-	-	10 (Plan 2)	
Group A	4	0.26	10.5	50	-	
Group B	4	0.26	10.5	1 k	10 (Plan 2)	
Group C	4	0.26	10.5	2 k	-	
Group D	4	0.32	10.5	50	-	
Group E	4	0.32	10.5	1 k	-	
Group F	4	0.32	10.5	2 k	-	

### Experimental procedures

The animals were anaesthetized by intraperitoneal injection of 2% pentobarbital sodium at 2 ml/kg, and the tumour surface was shaved and depilated. A catheter connected to a 22G needle was inserted into the caudal vein to establish the channel for intravenous injection. High-resolution two-dimensional (2-D) DUS was performed with the same VINNO70 system and the X4-12 L transducer to find the maximal dimension of the tumour section (Fig. 1). Then, a standard CEUS was conducted staying on the section using low MI contrast mode and an intravenous bolus injection of 0.15 mL SonoVue®. Ten minutes after the CEUS study, the hand-held transducer was placed in contact with the tumour surface but separated with a 2-cm-thick gel pad while the Vflash treatment was turned on for 10 minutes. The parameters for USMB treatment were different in each group (Table 1). During the USMB treatment, 0.4 mL of SonoVue® suspension was slowly and constantly injected into the caudal vein during the treatment. After treatment, CEUS performance was repeated twice on the same 2-D section, immediately and 4 hours later (Fig. 1B). The control group received only sham US exposure without MB injection.

For the Dox study, the experimental animals were treated with PNP of 0.26 MPa and PRF of 1.0 kHz combination based on previous results of the best tumour perfusion enhancement, while the control received sham US exposure. Three hours and 20 minutes after treatment, 10 mg/kg Dox solution (Meilun, Dalian, China) was injected through the tail vein (Fig. 1C).

### Tumour perfusion quantitation

The dynamic video clips of CEUS before treatment, immediately after treatment and 4 hours after treatment were analysed by the perfusion parametric imaging software of the machine. After manual drawing of the tumour borderline, the machine could automatically generate a time-intensity curve (TIC) of tumour contrast intensity, including the peak intensity (PI) and area under curve (AUC) data. The PI is the peak value of the TIC, and the AUC is integrated by the area under the TIC within 60 s starting from TIC elevation.

For the calculation of the tumour perfusion area rate, the images of the largest tumour contrast perfusion area in the clip were intercepted. Then, the tumour perfusion area was manually delineated using Adobe Photoshop CC (Adobe), and the rate of tumour perfusion area was calculated by the perfusion area/entire tumour area ×100%. The increment of the tumour perfusion area rate was calculated by the percentage of perfusion rate after treatment minus the percentage of perfusion rate before treatment.

### Vasodilators and inflammatory factors

Immediately after the experimental procedures, 56 animals in the perfusion study were sacrificed by inhalation of carbon dioxide with exposure to 100% CO_2_ at a filling rate of 20% cv/min. The tumours from Groups B and E and the control were harvested. The tumour tissues were minced into small pieces and homogenized. Then, the homogenates were centrifuged to obtain the supernatant for enzyme-linked immunosorbent assays (ELISAs).

The contents of eNOS, PGE2, PGD2, PGF2, PGI2, C3a, C5a, LTC4 and TNF-α in tumour tissues were determined by the Rat eNOS-3 ELISA Kit, Rat PGE2 ELISA Kit, Rat PGD2 ELISA Kit, Rat PGF2α ELISA Kit, Rat PGI2 ELISA Kit, Rat C3a ELISA Kit, Rat C5a ELISA Kit, Rat LTC4 ELISA Kit and Rat TNF-α ELISA Kit, respectively (MEIMIAN Industrial Co., Ltd., Jiangsu, China). The absorbance optical density (OD) of each well was measured at 450 nm. The levels of ATP, NO and ROS in tumour tissues were determined by an ATP assay kit, NO assay kit and reactive oxygen species assay kit (Nanjing Jiancheng Bioengineering Institute, China), and the OD values were measured by Microplate Reader according to the instructions.

### Dox concentration

For the quantification of the Dox concentration, the rats in the treated group (n = 14) and the control group (n = 11) were sacrificed 40 minutes after Dox infusion. Approximately half of the tumour bulk tissues were taken, and the Dox content was determined by HPLC.

Another half of the tumour sample was frozen and sliced, the nuclei were stained with DAPI, and the sections were examined under a fluorescence microscope (Nikon Eclipse C1, Nikon, Japan). Dox can spontaneously emit red light, while the nuclei appeared blue under UV excitation.

### Histological examination

One tumour sample from the treated group or the control was stained with haematoxylin and eosin (H&E) for morphological observation. Under a light microscope, tumour cells are surrounded by connective tissue in a disordered arrangement.

### Statistical analysis

SPSS 25.0 software was used for statistical analysis. Multifactor repeated-measures ANOVA was used to determine the influence of different groups on the blood perfusion of Walker-256 tumours at different time points for the PI, AUC and tumour perfusion area of CEUS. If there was an interaction, it was necessary to test the separate effects, and the Bonferroni method was used for pairwise comparison. The contents of ATP, eNOS, PGF2, PGI2, LTC4, TNF-α and ROS in tumour tissues were analysed by one-way ANOVA with a completely random design, and the LSD method was used for further comparison between groups. The variance of NO, PGE2, PGD2, C3a and C5a in tumour tissues was uneven. The independent sample Kruskal‒Wallis rank sum test was used, and Bonferroni correction was used for further comparison between groups. The concentration of Dox in tumour tissues was determined by an independent sample T test. A *p* value less than 0.05 was considered statistically significant.

## Results

### Tumour blood perfusion

**Fig. 2:**
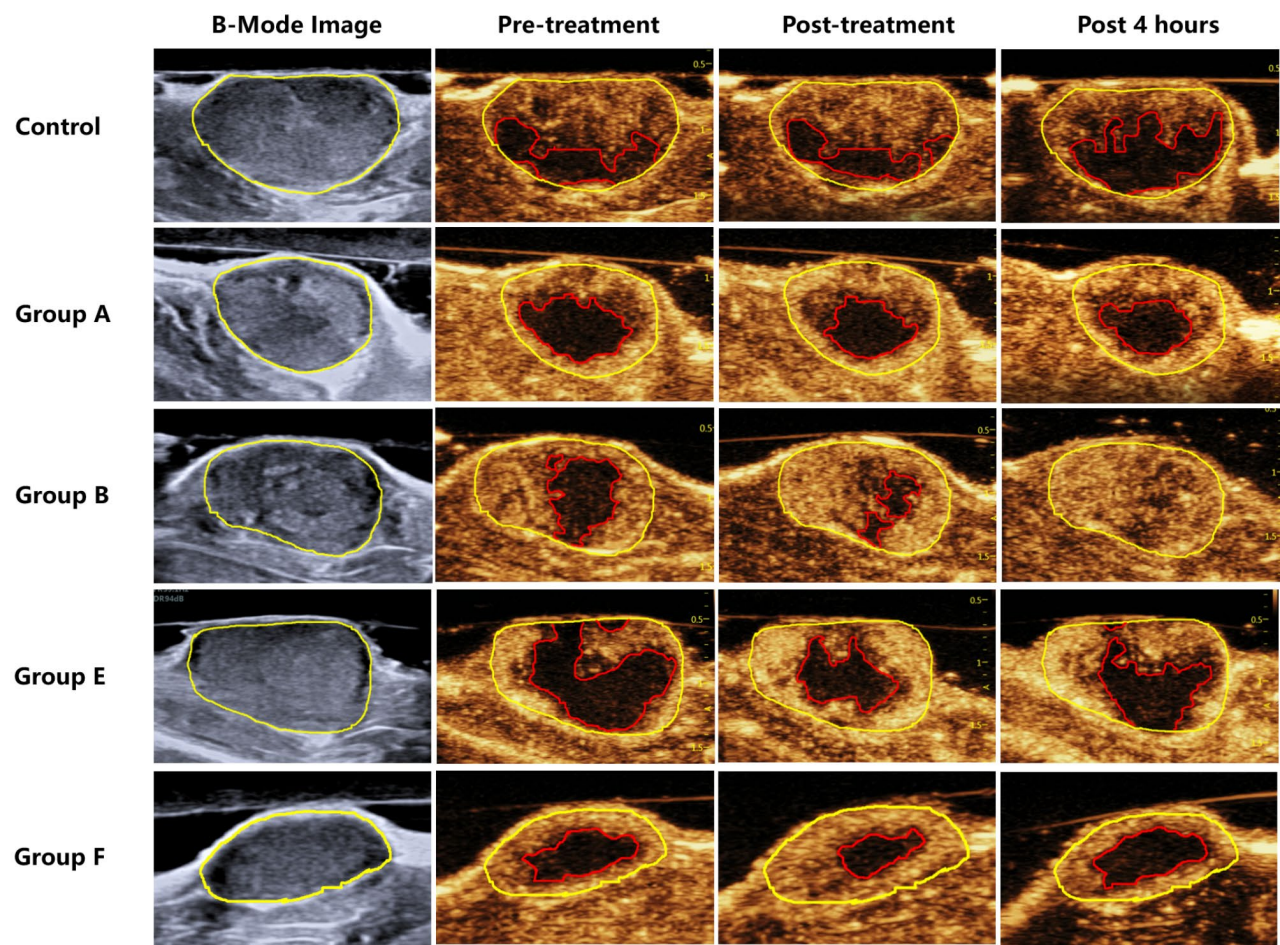
B-Mode and CEUS images of tumours in the five groups. Compared with Pre-treatment and Post-treatment, tumour perfusion increased significantly in Groups B and E and further increased after 4 hours in Group B. No significant perfusion change was found in the other groups

**Fig. 3:**
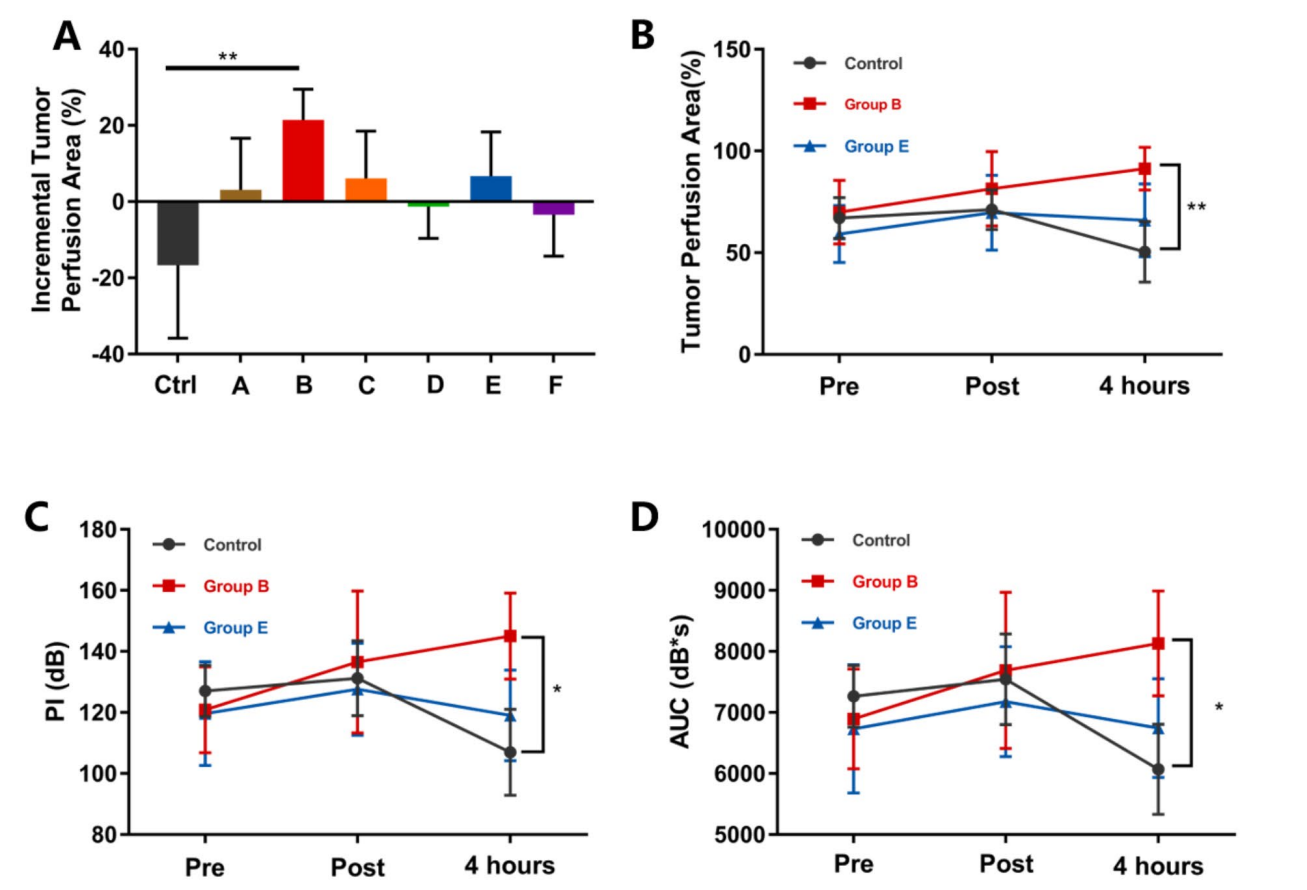
A: The percentage of tumour perfusion increased 4 hours after treatment compared with pre-treatment. In Group B, the incremental perfusion area was 21.42%. **P < 0.01. B, C, D: The variations in the perfusion area rate and the PI and AUC values of tumours in the control group and Groups B and E. *P < 0.05, **P < 0.01

The results showed that the sononeoperfusion effects of Groups B and E (PRF 1.0 kHz) were significant immediately after treatment (*P* < 0.05) (Tables 2 and 3). Immediately after treatment, the PI increased by an average of 12.39% in Group B and an average of 7.17% in Group E (P < 0.05). The AUC increased by an average of 11.11% in Group B and an average of 7.34% in Group E (*P* < 0.05). The average incremental perfusion area rate was 11.84% in Group B and 10.53% in Group E (*P* < 0.05). However, for the other groups (A, C, D, F and the control), the PI elevations ranged from 3.12 to 7.28%, the AUC from 2.81 to 7.33%, and the incremental perfusion area from 4.06 to 8.41%, and none of there were significant (*P* > 0.05). Four hours after treatment, the effect was further enhanced in Group B (PNP 0.26 MPa) with an increase of 20.29% in PI, 18.22% in AUC and 21.42% in incremental perfusion area when compared with the baseline (*P* < 0.05). There was no significant difference in Group E (PNP = 0.32 MPa) and the other groups 4 h later (*P* > 0.05) (Tables 2 and 3) (Figs. 2 and 3).

**Table 2:** PI and AUC values of CEUS before and after treatment ($\bar x \pm s$)

Group	PI (dB)				AUC (dB•s)		
	Pre-treatment	Post-treatment	4 h later		Pre-treatment	Post-treatment	4 h later
Control A B C D E F	127.1 ± 8.4 114.4 ± 11.6 120.9 ± 14.1 124.7 ± 22.4 113.2 ± 11.2 119.7 ± 17.0 118.1 ± 6.3	131.2 ± 12.3 119.0 ± 8.0 136.5 ± 23.3^a^ 132.9 ± 19.7 118.7 ± 16.3 127.6 ± 15.1^a^ 124.7 ± 9.6	106.9 ± 14.2^b^ 119.4 ± 23.3 145.0 ± 14.1^ac^ 125.2 ± 19.2 117.2 ± 14.6 119.0 ± 14.8 118.4 ± 10.6		7262.6 ± 505.5 6481.5 ± 646.3 6892.3 ± 817.1 7050.0 ± 1256.6 6384.1 ± 703.2 6730.4 ± 1049.6 6632.6 ± 427.4	7543.0 ± 740.3 6633.8 ± 439.8 7690.6 ± 1280.8^a^ 7508.1 ± 1105.7 6556.5 ± 669.2 7178.7 ± 899.9^a^ 6805.3 ± 652.6	6094.4 ± 779.4^ab^ 6811.7 ± 1260.8 8131.1 ± 858.5^ac^ 6972.5 ± 998.8 6446.1 ± 810.2 6743.5 ± 807.9 6665.1 ± 617.6

Compared with the same group before treatment, ^a^P < 0.05; compared with the same group immediately after treatment, ^b^P < 0.05; compared with the control at the same time, ^c^P < 0.05

**Table 3:** The percentages of perfusion area before and after treatment ($\bar x \pm s$)

Groups	Pre-treatment(%)		Post-treatment(%)		4 h later(%)
Control Group A Group B Group C Group D Group E Group F	67.12 ± 10.07 60.88 ± 15.46 69.94 ± 15.67 67.41 ± 12.84 58.08 ± 17.39 59.22 ± 14.10 68.94 ± 15.48		71.18 ± 9.76 66.91 ± 13.91 81.42 ± 18.26^a^ 75.82 ± 13.05 62.65 ± 19.12 69.75 ± 18.46^a^ 76.34 ± 13.36		50.42 ± 14.89^b^ 63.98 ± 22.82 91.36 ± 10.59^ac^ 73.50 ± 17.16 56.79 ± 20.30 65.94 ± 17.81 65.51 ± 19.21^b^

Compared with the same group before treatment, ^a^P < 0.05; compared with the same group immediately after treatment, ^b^P < 0.05; compared with the control at the same time, ^c^P < 0.05

### Dox concentration

**Fig. 4:**
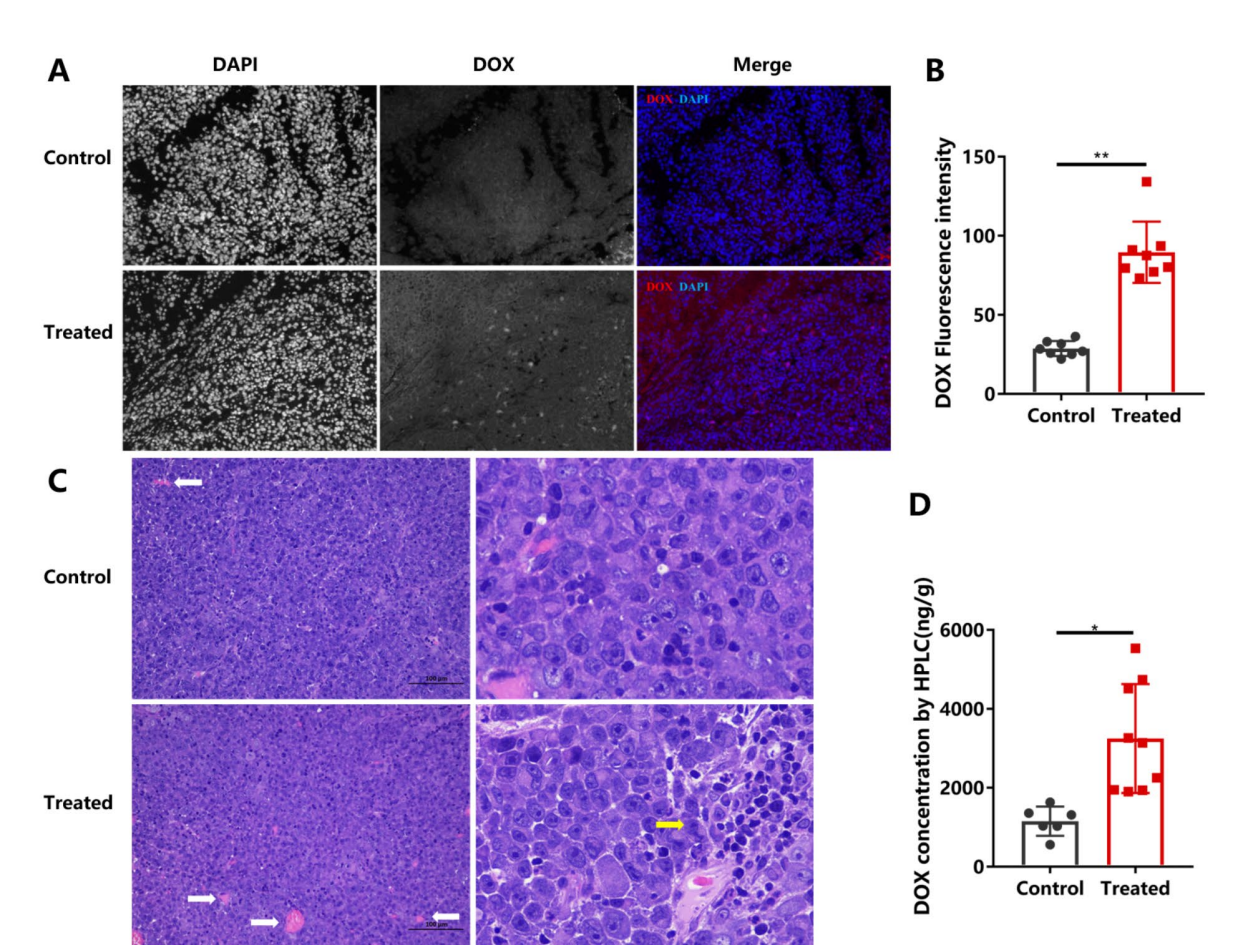
A, The Dox concentration in the control and treated groups was observed under a fluorescence microscope 4 hours after treatment. B, The mean fluorescence intensity of Dox was significantly higher in the treated group than in the control group. C, Four hours after treatment, HE sections showed microvascular hyperaemia (white arrow) and inflammatory cell infiltration (yellow arrow) in the treated tumours but not in the control tumours. D, The Dox concentration was significantly higher in the treated group than in the control by HPLC. *P < 0.05

Four hours after treatment, we applied HPLC and fluorescence microscopy to evaluate the Dox concentration within the tumour tissues. HPLC showed that the Dox concentration in the control group was 1152.71 ± 369.83 ng/g and that in the treated group (PNP 0.26 MPa, PRF 1.0 kHz) was 3246.59 ± 1301.85 ng/g. The Dox concentration in the treated tumours was 2.82-fold higher than that of the control (Fig. 4D). Fluorescence microscopy showed that Dox fluorescence intensity in the treated group (PNP 0.26 MPa, PRF 1.0 kHz) was significantly higher with a wider distribution compared with that of the control (Fig. 4A). The average Dox fluorescence intensity of the treated group was 3.12-fold greater than that of the control (Fig. 4B).

### Cytokine detection and histological examination

**Fig. 5:**
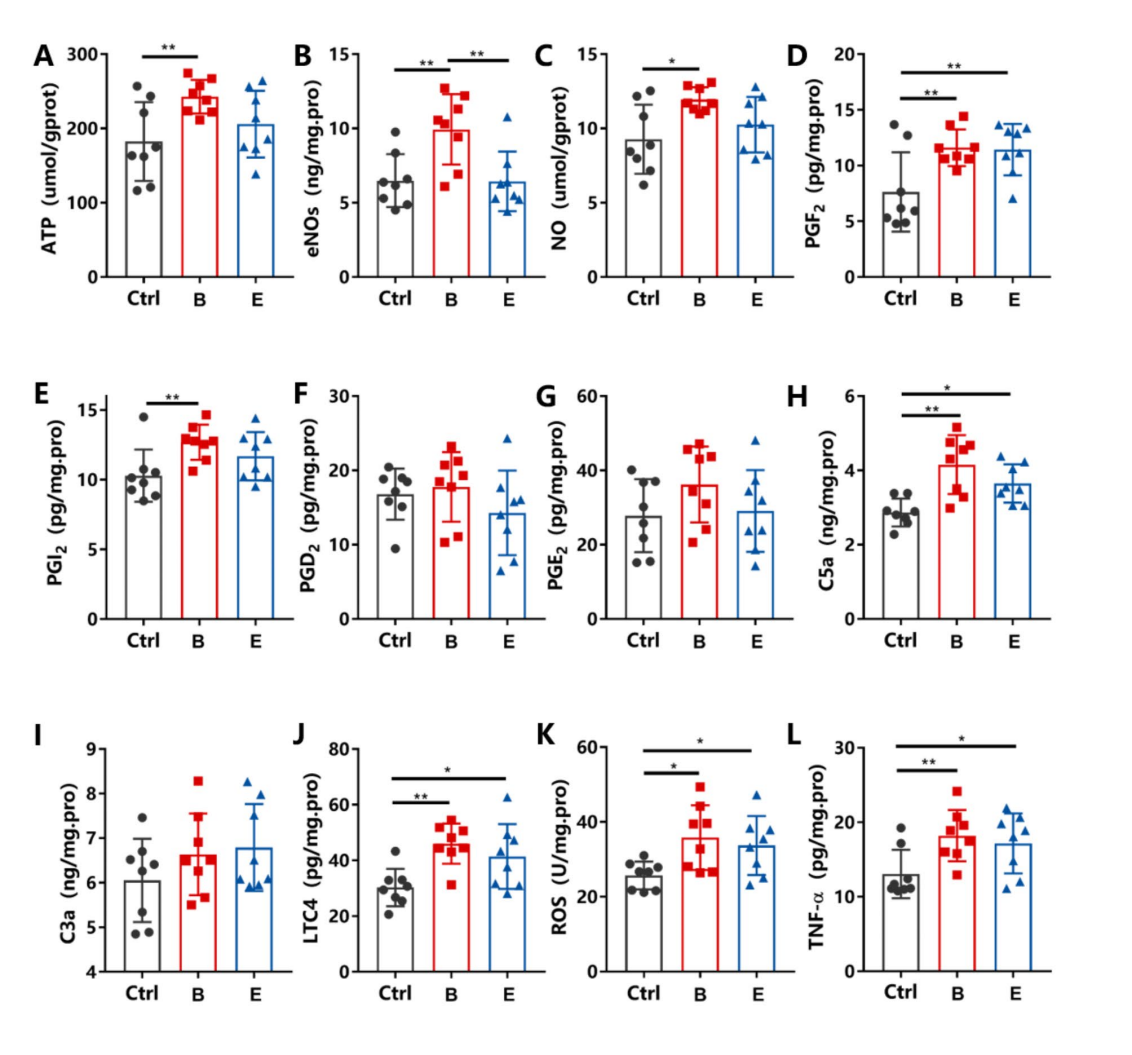
A-L, The contents of ATP, eNOs, NO, PGF2, PGI2, PGD2, PGE2, C5a, C3a, LTC4, ROS and TNF-α in tumour tissues of the control, Group B and Group E 4 hours after treatment. *P < 0.05, **P < 0.01

ELISAs showed that cytokines, including vasodilators and inflammatory factors, increased 4 hours after treatment. ATP, eNOS, NO, PGF2, PGI2, C5a, LTC4, TNF-α and ROS in Group B (PNP 0.26 MPa, PRF 1.0 kHz) were higher than those of the control (*P < 0.05*). There were no significant changes in PGD2, PGE2, or C3a among the groups (*P > 0.05*) (Fig. 5).

Light microscopy revealed that microvascular hyperaemia and inflammatory cell infiltration were obvious (Fig. 4C) 4 hours after treatment in the treated tumours (PNP 0.26 MPa, PRF 1.0 kHz) (*P < 0.05*), while there was no significant difference in the control (*P > 0.05*).

## Discussion

In this study, we comprehensively investigated the proper acoustic parameters, duration time, perfusion area, Dox delivery, cytokines and related pathological changes associated with the sononeoperfusion effect. For the first time, we proposed the new term “sononeoperfusion” to represent this tumour perfusion enhancement effect induced by DUS and microbubbles. The effect had previously appeared in immunotherapy of MC38 colon cancer and chemotherapy of PANC-1 pancreatic cancer in mice [20, 21]. This study further explored the effect from many aspects mentioned above to obtain a better understanding.

First, therapeutic US was generated from a modified DUS system (VINNO 70) as previously described [24]. The system was modified with a new Vflash mode based on its conventional flash mode, that is, a microbubble destruction mode during CEUS. The acoustic emission can be weakly focused to a ROI by using electronic phased-focus technology, unlike the small and strong focus of high-intensity focused US. Furthermore, the Vflash mode can regulate cavitation by changing the MI, PL, PRF and destruction/replenishment time [24]. Additionally, different from some previous studies using B-mode [26] or conventional flash mode [27], the Vflash mode is not only able to provide sufficient microbubbles as cavitation nuclei during replenishment but can also weakly focus the cavitation activities to a designated ROI and regulate the cavitation intensity for microbubble vibration or destruction. All US emissions were confined to FDA and IEC guidelines.

Second, the sononeoperfusion effect was remarkable and repeatable under proper USMB treatment, i.e., only in Groups B and E. The best tumour perfusion improvement was observed in Group B (PNP 0.26 MPa and PRF 1.0 kHz) with a 20.29% increase in PI, an 18.22% increase in AUC and a 21.42% increment in the perfusion area rate (Tables 2 and 3). This effect lasted for 4 hours. Previous studies determined that sonoporation had a significant therapeutic effect when using a long PL, specifically 40-µs pulses [28, 29]. To explore the variations in PNP and PRF, we selected a burst of 10.5 cycles as the PL within the limitation of regulation instead of 1–2 cycles of conventional DUS. Obviously, 1.0 kHz was the best PRF in this study to stimulate tumour perfusion (Fig. 2), and 50 Hz and 2.0 kHz might be either too low or too high in acoustic intensity, thereby failing to induce the effect. PNP is regarded as the most related parameter in cavitation [30], and a low PNP amplitude of 0.26 MPa was preferable to acquire the effect (Tables 2 and 3). The 0.32 MPa PNP seemed to be less effective. The incremental percentage of the tumour perfusion area rate, which might be the most convincing evidence of the sononeoperfusion effect, was 11.48% immediately after 0.26 MPa USMB treatment and continually rose to 21.42% four hours later. For 0.32 MPa USMB, the increment was 10.53% immediately after but dropped to 6.72% four hours later. This result indicated that the effect required low PNP under 0.3 MPa and a proper PRF of 1.0 kHz. Stable cavitation usually dominates under 0.4 MPa PNP [31]. Therefore, the sononeoperfusion effect was likely to be linked with microbubble stable cavitation. These results were consistent with some previous studies on US drug delivery using low pressure below 0.4 MPa [31]. It is obvious that low PNP means less cavitation bioeffects or less risk in clinical translation.

Third, US-mediated drug delivery has been well documented in many studies [31]. The USMB treatment combination of 0.26 MPa and 1.0 kHz demonstrated not only the best perfusion effect but also resulted in good Dox delivery. HPLC and fluorescence microscopy showed that the Dox concentration of the treated tumours was up to 3.12-fold higher than that of the control (Fig. 4B and D). This means that the simple combination of usual DUS, intravenous administration of Dox and SonoVue® microbubbles may provide a convenient way to gain a better chemotherapeutic effect, as in a clinical pancreatic cancer study [32]. Previous studies have always attributed USMB-enhanced drug delivery to sonoporation [28, 33], a process in which US activates microbubbles and increases the permeability of biological barriers [34]. However, the sononeoperfusion effect might be another effect existing in USMB-enhanced drug delivery, which has been ignored by other related studies. We use the designation USMBs here instead of ultrasound-targeted microbubble destruction (UTMD), a term strongly connected to inertial cavitation.

It is well known that solid tumours always develop hypoperfused and hypoxic areas, resulting in chemotherapy, radiotherapy and immunotherapy resistance. This hypoxic area lies between the perfused tumour and necrotic tumour [35, 36]. We consider that the sononeoperfusion effect might stimulate and recover the blood perfusion of the area, thus increasing the tumour perfusion area and improving drug delivery.

Finally, we tried to explain the mechanism of sononeoperfusion by detecting related cytokines within tumour tissues. The mechanical effect of stable cavitation under 0.3 MPa [33] may release microstreaming and shear force. These mechanical effects permeabilize the vascular wall, called sonoporation, but they also cause slight injury to the wall. The injury could trigger an inflammatory response and the repair process. The inflammatory response triggers vasodilation and an increase in vascular permeability through the release of cytokines. ELISAs showed that cytokines were significantly increased, such as vasodilators, ATP, eNOS, NO, PGF2, and PGI2, as well as inflammatory factors, including C5a, LTC4, and TNF-α (Fig. 5). Light microscopic manifestation also supported the inflammatory response in that microvascular hyperaemia and inflammatory cell infiltration were observed in the USMB-treated tumour (Fig. 4C). Since the USMB at a stable cavitation level can only produce minor mechanical injury to the vessel wall, it cannot cause significant changes in microscopic tumour morphology. Furthermore, ROS, which are oxygen-containing molecules with high reactivity, can reduce multidrug resistance and initiate oxidative stress-induced tumour cell death [37]. ROS were overproduced 4 hours after USMB treatment.

Sonoreperfusion effects have been discovered in skeletal muscle in recent years and may be a promising solution for peripheral vascular diseases or muscular ischaemia [18]. Since sonoreperfusion can only be stimulated under microbubble inertial cavitation with a high PNP of 0.9–1.7 MPa [18], the sononeoperfusion effect is likely to be induced only under stable cavitation with PNP ranging from 0.26 to 0.32 MPa. The noninvasive sononeoperfusion effect, operating within the diagnostic intensity, might be a novel physical method to overcome hypoperfused or hypoxic conditions of solid tumours that are confirmed to have therapeutic resistance. Another possible application of sononeoperfusion would be a quick prediction of therapeutic response once it is confirmed to be connected with hypoxic tumours. The only potential risk of this effect would be tumour metastasis, and the risk has been proven negative in our previous study [24]. The effect might have existed in many previous related studies but was neglected [26, 32].

This is a preliminary experimental study. We did not test more parameters or measure the cavitation magnitude for the sononeoperfusion effect, considering the complexity of cavitation. Proper acoustic parameter combinations, including the microbubble concentration, may greatly influence the effect. This study did not prove the improvement in the hypoxic microenvironment of solid tumours. In addition, the mechanistic study of the sononeoperfusion effect was only limited to the inflammatory response. Further signalling molecules and pathways related to the effect should be taken into consideration.

## Conclusion

In this work, we demonstrated that modified DUS combined with microbubbles enhances blood perfusion of rat Walker-256 tumours, which was named the sononeoperfusion effect, thus promoting chemotherapeutic drug (Dox) delivery by up to 3.12-fold. This study also demonstrated that the sononeoperfusion effect might be related to the inflammatory response by the release of vasodilators and inflammatory factors.

## Electronic supplementary material

Below is the link to the electronic supplementary material.

Supplementary Material 1

Supplementary Material 2

## Data Availability

All data generated or analysed during this study are included in this published article.

## Abbreviations

AUC: area under curve
CEUS: contrast-enhanced ultrasound
Dox: doxorubicin
DUS: diagnostic ultrasound
ELISA: immunosorbent assay
H&E: haematoxylin and eosin
HPLC: high-performance liquid chromatography
MI: mechanic index
OD: optical density
PI: peak intensity
PL: pulse length
PNP: peak negative pressure
RF: pulse repetition frequency
ROI: region of interest
SD: Sprague‒Dawley
TIC: time-intensity curve
US: ultrasound
USMB: ultrasound stimulated microbubble
UTMD: ultrasound-targeted microbubble destruction
