# Supplementary material for: Sononeoperfusion: a new therapeutic effect to enhance tumour blood perfusion using diagnostic ultrasound and microbubbles
Source: Cancer Imaging. 2023 Mar 23;23:29. doi: 10.1186/s40644-023-00545-y (PMC10035258; doi:10.1186/s40644-023-00545-y)
Supplement: Supplementary file 2 — Supplementary Material 2 [file 40644_2023_545_MOESM2_ESM.pdf]

SPRINGER NATURE

Author Services

# Editing Certificate

This document certifies that the manuscript

## Sononeoperfusion: A new therapeutic effect to enhance tumour blood perfusion using diagnostic ultrasound and microbubbles

prepared by the authors

Najiao Tang†, Jiawei Tang†, Junhui Tang, Qiong Zhu, Xiaoxiao Dong, Yi Zhang*, Ningshan Li, Zheng Liu*

was edited for proper English language, grammar, punctuation, spelling, and overall style by one or more of the highly qualified native English speaking editors at SNAS.

This certificate was issued on **December 30, 2022** and may be verified on the SNAS website using the verification code **CFFE-03A8-BFE1-9DAD-93C7**.

Neither the research content nor the authors' intentions were altered in any way during the editing process. Documents receiving this certification should be English-ready for publication; however, the author has the ability to accept or reject our suggestions and changes. To verify the final SNAS edited version, please visit our verification page at secure.authorservices.springernature.com/certificate/verify.
If you have any questions or concerns about this edited document, please contact SNAS at support@as.springernature.com.

SNAS provides a range of editing, translation, and manuscript services for researchers and publishers around the world.
For more information about our company, services, and partner discounts, please visit authorservices.springernature.com.
