# Supplementary material for: Sononeoperfusion: a new therapeutic effect to enhance tumour blood perfusion using diagnostic ultrasound and microbubbles
Source: Cancer Imaging. 2023 Mar 23;23:29. doi: 10.1186/s40644-023-00545-y (PMC10035258; doi:10.1186/s40644-023-00545-y)
Supplement: Supplementary file 1 — Supplementary Material 1 [file 40644_2023_545_MOESM1_ESM.docx]

Dear editor,

RE: Manuscript “Sononeoperfusion: A new therapeutic effect to enhance tumour blood perfusion using diagnostic ultrasound and microbubbles” (CAIG-D-22-00263)

We would like to thank Cancer Imaging for giving us an opportunity to revise our manuscript.

We thank Associate Editor and Referees for reviewing our paper. We have carefully taken their comments into consideration and made a point-by-point response.

The manuscript has been edited by Nature Research Editing Service. The Editing Certificate was added and listed at the end.

Below is our response to their comments.

Thanks for considering our manuscript. If you have any questions, contact us at liuzhengs@hotmail.com.

Sincerely,

Dr. Liu

Corresponding Author

---------------------------------------------------------------------------------------------------------------------------------

Response to editor:

Reviewer #1: For animal experiments, explicit approval procedures are required in the EU and especially because of CEUS with SonoVue, since these are off-label use applications. The research approaches already carried out by Lassau N, Weg A, Bitterre F and Hackl C, published in Ultrasound in Med and the EFSUMB guidlines, among others, must be considered and discussed. The possibility of CEUS perfusion imaging with the VueBox should be considered. It is important to pay attention to references to MRI perfusion and diffusion, alternatively in the animal model. SonoVue is not tumor specific. For example, there would be BR55. This must be taken into account.

Thank you for the comments.

All of the animal experimental procedures, including the diagnostic or therapeutic use of SonoVue in CEUS, were approved by the Institutional Animal Care and Use Committee of the University. Here in this study, the tumor perfusion enhancement effect, i.e., the therapeutic effect of US and microbubble, was our main concern or focus. As we know, this effect has not been reported or filed in any literature or patent yet.

Since CEUS is a reliable method to assess the tumor perfusion, so it was used in this study. In order to emphasize this point, we had added *“CEUS is a reliable method to assess tumour perfusion. According to the EFSUMB guidelines, the mean time-intensity curves within the tumours after bolus injection of a contrast agent were qualified to reflect microbubble wash-in and washout, thus representing the condition of tumour perfusion.”* in the Methods section [1]. (Page 6, Line 13-17)

VueBox™ is a quantification toolbox designed to analyze dynamic CEUS DICOM clips. The motion correction of VueBox™ enables a more precise evaluation concerning moving wash-in and wash-out kinetics. In this study, we performed CEUS on the subcutaneous tumors in rats, and the tumors were almost motionless. The integrated contrast software (Contrast Bubble Imaging, CBI) was able to quantify data as effective as VueBox™, like the integrated softwares in the other machines (QLAB for Philips, and TIC Analysis for GE).

As mentioned above, CEUS is a reliable imaging method to assess tumor perfusion. The US machine used in this study can provide both CEUS and low-intensity US treatment conveniently at the same time. MRI perfusion and diffusion demands the availability of MRI facility and takes longer time to perform the perfusion assessment. We will consider MRI perfusion, photoacoustic imaging and other methods to prove the US perfusion effects in our future works.

BR55 is an experimental, targeted contrast agent for VEGFR2 which is highly expressed in neovascular endothelial cells. But in this study, we are looking for the perfusion changes of tumor after USMB treatment, not for tumor differential diagnosis. So, tumor specific contrast imaging is not necessary.

*References:*

*1.	Sidhu, P.S., et al., The EFSUMB Guidelines and Recommendations for the Clinical Practice of Contrast-Enhanced Ultrasound (CEUS) in Non-Hepatic Applications: Update 2017 (Long Version). Ultraschall Med, 2018. 39(2): e2-e44.*

Reviewer #2: In this work, researchers demonstrated that combined with microbubbles enhances blood perfusion, discovered the effective parameter, with PNP 0.26 MPa and PRF 1.0 kHz. And this effect could last 4 hours with good Dox delivery, promoting chemotherapeutic drug (Dox) delivery by up to 3.12-fold. This is of great significance for the treatment of tumor with deficient blood supply. In future research, the parameters can be refined to find a more accurate rang. This study also demonstrated that the sononeoperfusion effect might be related to inflammatory response by the release of vasodilator and inflammatory factors.

However, there are some minor problems that need to be confirmed by the author. Firstly, P6-line6 at 3h and 20min or at 3h20min? Secondly, in Table 3, data in column"4 hours later" is not in the correct location. Thirdly, whether there has error by PS manual delineation perfusion rate, how to reduce or eliminate this manual delineation error?

Thank you for your comments.

Firstly, we have corrected the expression error in Figure 1 and its caption on P5. We replaced “3 hours and 20 minutes” with “3 hours 20 minutes”.

Secondly, data in column"4 hours later" is now in the correct location in Table 3.

Thirdly, yes, there would be errors by manual delineation since the perfusion rate depends highly on manual draw of tumor borderline. To reduce or eliminate this manual error, all of the tumor delineation was performed by the same doctor experienced in CEUS. And the results of perfusion area (Fig. 3B) were consistent with that of PI and AUC values (Fig. 3CD), which might exhibit the reliability of the manual delineation method.
